# The Effect of Green Tea as an Adjuvant to Enzyme Replacement Therapy on Oxidative Stress in Fabry Disease: A Pilot Study

**DOI:** 10.3389/fnut.2022.924710

**Published:** 2022-07-08

**Authors:** Giovanni Bertoldi, Gianni Carraro, Verdiana Ravarotto, Valentina Di Vico, Paola Baldini Anastasio, Nicola Vitturi, Francesco Francini, Lucia Federica Stefanelli, Lorenzo A. Calò

**Affiliations:** ^1^Nephrology, Dialysis and Transplantation Unit, Department of Medicine, University of Padova, Padua, Italy; ^2^Metabolic Diseases Unit, Department of Medicine, University of Padova, Padua, Italy; ^3^Clinical Nutrition Unit, Department of Medicine, University of Padova, Padua, Italy

**Keywords:** fabry disease, oxidative stress, green tea, epigallocatechin-3-gallate (EGCG), enzyme replacement therapy (ERT)

## Abstract

Enzymatic replacement therapy (ERT) is not very effective in halting the progression of Fabry disease (FD) toward cardiovascular (CV)-renal remodeling, particularly in case of late diagnosis. FD patients have increased oxidative stress (OS), critical for the induction of CV-renal remodeling. We investigated the effects of an adjuvant antioxidant treatment to ERT on OS and the possible advantages for related complications. OS was evaluated in 10 patients with FD before ERT, after 12 months of ERT, and after 6 months of adjuvant green tea (GT) to ERT by the following experiments: expression of p22^phox^; phosphorylation state of MYPT-1 and ERK 1/2 (by western blotting); and quantification of malondialdehyde (MDA) and heme oxygenase (HO)-1 levels (by ELISA). p22^*p*hox^ and MYPT-1 phosphorylation decreased after ERT and significantly further decreased after GT. ERK 1/2 phosphorylation and MDA levels remained unchanged after ERT, but significantly decreased after GT. HO-1 significantly increased after ERT and further increased after GT. This study provides preliminary data highlighting the antioxidant effect exerted by ERT itself, further amplified by the adjuvant antioxidant treatment with GT. The results of this study provide evidence of the positive effect of early additive antioxidant treatment to reduce OS and prevent/alleviate cardio and cerebrovascular-renal complications related to OS.

## Introduction

Fabry disease (FD, OMIM 301500) is an X-linked rare metabolic condition due to mutations in the alpha galactosidase (*GLA*) gene that encodes for the lysosomal enzyme, alpha-galactosidase A (α-GalA). The resulting reduced or altered enzymatic activity causes progressive accumulation of glycosphingolipids, mainly globotriaosylceramide (Gb3) and its deacetylated form lyso-Gb3, within lysosomes leading to the onset of a systemic storage disorder (1). FD is pan-ethnic disease; however, because of its rare nature and complex clinical characteristics, the true prevalence of FD may be largely underestimated. The most widely reported incidence ranges between 1:40,000 and 1:117,000 in the general population (2, 3).

Early onset symptoms with neurological manifestations such as acroparesthesia and intense limb pain generally appear during childhood, and typically decrease in frequency with growth (4). Other common manifestations at a young age include gastrointestinal disorders (diarrhea, pain, postprandial bloating, nausea); hypohidrosis; angiokeratomas; and ocular lesions. FD has a progressive course and by 30–40 years of age, the disease occurs with severe implications that include the renal (proteinuria, renal failure, hypertension); cardiovascular (left ventricular hypertrophy, arrhythmias, heart failure); and neurological (transient ischemic attacks, stroke) systems, with a progressive decline in the quality of life (5). The overlap of symptoms with more common diseases is the main cause of delayed diagnosis in FD, and patients often remain undiagnosed for years (6).

The current available treatment for all FD patients is enzyme replacement therapy (ERT), which comprises the intravenous administration of recombinant α-GalA to replace the impaired enzymatic activity and reduce intracellular storage of Gb3. Short-term studies on the effect of ERT have reported a reduction in left ventricular mass (7), as well as a decrease in neuropathic pain and an overall improvement in cardiac and renal outcomes (8). Nevertheless, long-term studies have shown that ERT can prevent the risk of developing complications, but does not modify the natural course of cardiac, cerebrovascular, and renal diseases (9, 10). Hence, for an optimal clinical management of the renal, cardiac, neurological, and other complications of FD-induced chronic tissue injury, the expert recommendation is to combine ERT with supportive interventions (11). These observations suggest that the adverse FD outcomes do not entirely depend on the single accumulation of glycosphingolipids and that additional therapies targeted at specific secondary mechanisms might halt the progression of cardiac, cerebrovascular, and renal diseases that occur in FD patients.

Oxidative stress (OS) is widely considered to be involved in cardiovascular and renal complications (12). In a previous study (13), we clearly demonstrated with a molecular biology approach that OS is activated in FD patients and associated with the induction of cardiovascular remodeling. This evidence therefore suggests that in addition to ERT, OS inhibition by both pharmacological and nutritional measures may be useful in the prevention/treatment of cardiovascular-renal remodeling in FD. Previous studies by our team have shown that treatment with green tea (GT) significantly reduced the level of OS and related cell signaling mechanisms in another population with a high cardiovascular risk such as dialysis patients (14). In particular, GT had a positive influence on cardiovascular and renal remodeling and endothelial function of these patients as shown after 6 months of treatment (14).

Green tea contains high amount of flavonoids such as catechins and has been shown to have high antioxidant property *in vitro*, *in vivo* in animal models, and in clinical trials in humans (15). Furthermore, GT plays a role in risk reduction of several chronic diseases such as neuroinflammation, cardiac dysfunction, renal damage, and atherosclerosis (16–19).

Among GT catechins, epigallocatechin-3-gallate (EGCG) is the most abundant and biologically active and can induce autophagy, anti-inflammatory action via degradation of lipid droplets in endothelial cells, and facilitate the degradation of endotoxins (20). In addition, EGCG has been shown to exert anti-inflammatory function in several animal models of acute kidney injury, glomerulonephritis and lupus nephritis, diabetic nephropathy, and renal fibrosis (21). *In vitro* experiments showed the efficacy of EGCG in reducing TNFα, IL-1β, IL-6, and inducible nitric oxide synthase (iNOS) via inhibition of ROS-dependent activation of NF-κB and in increasing nuclear erythroid-2 related factor 2 (Nrf2)/HO-1 antioxidant signaling (22).

It is therefore reasonable to think that the administration of GT as adjuvant therapy to ERT in FD patients might have positive effects on OS, inflammation, cardiovascular-renal remodeling, and impaired autophagy.

In this study, we evaluated the effect of ERT itself on the altered OS status in FD, and assessed both the effect of ERT alone and that of adjuvant GT on the activated biochemical pathways related to OS signaling. Specifically, we evaluated the protein expression of p22^*p*hox^ (subunit of NADH/NADPH oxidase essential for the induction of OS); the phosphorylation state of MYPT-1, marker of Rho kinase activity (which is deeply involved in the induction of OS and cardiovascular-renal remodeling); and phosphorylation state of ERK 1/2 (effector of OS for cardiovascular-renal remodeling). Plasma levels of malondialdehyde (MDA) and heme oxygenase (HO)-1 were also evaluated.

## Materials and Methods

### Design of the Study

**FIGURE 1:**
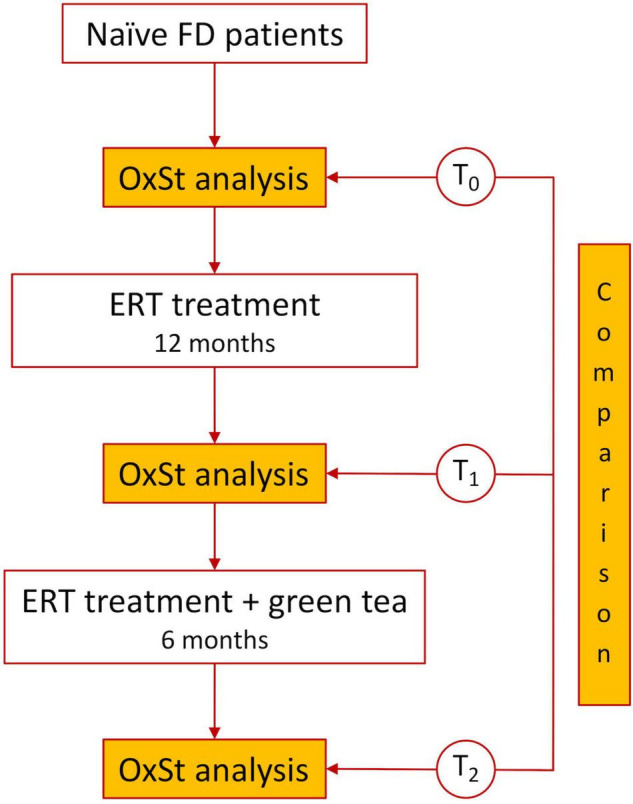
Flow chart of the study.

Ten FD naïve patients with a genetic and clinical diagnosis but not yet on ERT were enrolled in this study. This sample size ensured a statistical power of 90% with an alpha of 0.05 to detect a 20% reduction in p22^phox^ protein expression after 6 months of GT treatment. The sample size was calculated assuming that the mean ± SD of p22^phox^ protein expression is 1.76 ± 0.76 d.u. (14) and a 10% loss to follow-up. Circulating mononuclear cells were separated and the plasma obtained from patients’ baseline blood sampling (T_0_). Patients then started ERT and after 12 months, they had the second blood sampling (T_1_) before starting the additional treatment with two capsules of GT (*Camellia Sinensis*, 600 mg of leaves dry extract) taken in the morning in a fasted state, followed by the final blood sampling (T_2_) obtained after 6 months of GT therapy. The levels of specific biomarkers of OS at T_0_, T_1_, and T_2_ were evaluated and compared (Figure 1).

Ten FD naive patients (five male and five female, age range: 28–78 years, mean 52 ± 16), followed-up at the Nephrology Unit of the Padova University Hospital were enrolled according to the following inclusion and exclusion criteria. Inclusion criteria were a confirmed genetic and biochemical diagnosis of FD and not on ERT. The exclusion criteria were diabetes mellitus, cancer, heart failure, respiratory failure, liver failure, and any ongoing infections. The genetic characterization and the Lyso Gb3 levels of our cohort at T_0_ are shown in Table 1. None of the patients were smokers, and all abstained from food, alcohol, and caffeine-containing beverages at least 12 h prior to blood sampling.

**TABLE 1:** Genetic characterization and plasma levels of Lyso Gb3 assessed at baseline (T_0_) in our cohort of Fabry patients.

Patient nr.	Gender	G LA Mutation	AA substitution	Lyso Gb3 ng/mL
#1	Female	c.A644G	p.Asn215Ser	2, 9
#2	Male	c.A644G	p.Asn215Ser	5, 3
#3	Female	c.A644G	p.Asn215Ser	4, 8
#4	Male	c.A644G	p.Asn215Ser	4, 1
#5	Female	c.A644G	p.Asn215Ser	1, 2
#6	Male	c.A644G	p.Asn215Ser	3, 1
#7	Female	c.A644G	p.Asn215Ser	1, 9
#8	Female	c.1077delT	p.Ile359fs *	4, 7
#9	Male	c.835C > G	p.Gln279Glu	2, 7
#10	Male	c.835C > G	p.Gln279Glu	3, 3

*Plasma Lyso Gb3 normal levels are: ≤ 1.8 ng/mL.*

*Fs, frame shift.

Patients at the enrollment had normal kidney function in terms of eGFR (> 90 mL/min/1.73 m^2^, CKD-EPI) and serum creatinine levels. They also had normal lipid and glycemic profile. Patients’ blood pressure ranged from 110/70 to 140/90 mmHg. All patients were treated with algasidase-alpha (Shire HGT, Inc., Cambridge, MA, United States) 0.2 mg/kg every 2 weeks.

After 12 months (T_1_), patients were additionally treated with two capsules of commercially available GT (TE’ VERDE, Frama SRL, Noventa Padovana, Padua, Italy) taken in the morning in a fasted state. Two capsules contain 600 mg of *Camellia sinensis* leaves dry extract, of which 570 mg are total polyphenols (95%). Among polyphenols, 390 mg are catechins (65%), of which 240 mg (40%) are EGCG.

Each patient received one box of GT after T_1_ containing 60 capsules, needed for 30 days of treatment; the supply was dispensed after the due check. A standardized capsule formulation of GT was chosen to assure dosage and patients’ compliance to treatment. Adherence to treatment was ascertained by questioning the subjects regarding treatment adherence and diligence during their hospital visits every 2 weeks.

During the initial medical examination, patients were informed about the study protocol, and they provided informed written consent to participate in the study. The study was approved by the Ethics Committee of Padova University Hospital (CESC), protocol number 60829, 14/10/2020.

### Preparation of Mononuclear Cells

Blood samples were collected and processed the same day. Plasma fractions were isolated by centrifugation from 35 mL of EDTA-anticoagulated blood at room temperature and immediately stored at –80°C for the following procedures. Peripheral blood mononuclear cells (PBMCs) were isolated at room temperature by Lympholyte-H gradient (Cedarlane, Hornby, Ontario, CA). Total protein extracts were obtained by cell lysis using an ice-cold buffer (Tris-HCl 20 mmol/l, NaCl 150 mmol/l, EDTA 5.0 mmol/l, Niaproof 1.5%, Na_3_VO_4_ 1.0 mmol/l, SDS 0.1%) added with protease inhibitors (Roche Diagnostics, Mannheim, DE) and phosphatase inhibitors (Phosphatase Inhibitor Cocktail 1, Sigma-Aldrich, Saint Louis, MI, United States). Once collected, all samples underwent a three-cycle sonication at 60 mA. Protein concentration was quantified by BCA protein assay (Pierce, Rockford, Illinois, United States) at room temperature. The samples were stored at –80°C until further use.

### p22^phox^, ERK1/2, and MYPT-1 Western Blot Analysis

Protein expression of p22^phox^ and phosphorylation state of MYPT-1 and ERK1/2 were assessed by western blot analysis. Total protein lysates from patients were separated by SDS-PAGE in Tris pH 8.3 with a molecular weight marker (Full-Range Rainbow Molecular Weight Marker, GE Healthcare, Amersham Pl, United Kingdom). Gels were loaded with the same amount of sample. Proteins were transferred on nitrocellulose membranes using a Hoefer TE 22 Mini Tank Transphor Unit (Amersham Pharmacia Biotech, Uppsala, SE) in a buffer: 39 mmol/l glycine, 48 mmol/l Tris base, 0.037% SDS (electrophoresis grade), and 20% methanol at 350 mA for 2 h. After blocking with 5% BSA in Tween-PBS, each membrane was incubated overnight with a primary polyclonal antibody for the detection of target proteins: anti-p22^phox^ (Santa Cruz Biotechnologies, Santa Cruz, CA, United States); anti-MYPT-1 (Cell Signaling Technology, Danvers, MA, United States); anti-phospho MYPT-1 (Cell Signaling Technology); anti-ERK1/2 (Cell Signaling technology); and anti-phospho ERK1/2 (Cell Signaling Technology). Membranes were incubated with specific HRP-conjugated secondary antibodies; immunoreactive proteins were visualized with chemiluminescence using SuperSignal WestPico Chemiluminescent Substrate (Pierce, Rockford, IL, United States), and images were acquired with Amersham Imager 600 (Amersham Pharmacia Biotech, Uppsala, SE). To normalize the result, anti-monoclonal housekeeping antibodies such as α-tubulin (anti-α-tubulin, Santa Cruz Biotechnologies, Santa Cruz, CA, United States) and β-actin (Sigma-Aldrich, Saint Louis, MI, United States) were used. Specifically, α-tubulin was used to normalize the results of ERK, phospho ERK, MYPT-1, and phospho MYPT-1, while β-actin was used for normalizing the values of p22^phox^.

Western blot products were quantified by a densitometric semiquantitative analysis using NIH Image software (NIH, Rockville Pike Bethesda, MA, United States). The densitometric quantification of the targeted proteins were normalized by the densitometric quantification of the housekeeping protein observed in the same membrane.

### Lipid Peroxidation

Lipid peroxidation was evaluated in plasma samples in terms of MDA production, using the commercially available Lipid Peroxidation (MDA) Assay Kit nr. ab118970 (Abcam, Cambridge, United Kingdom). The assay was performed according to the protocol, along with the optional step for enhanced sensitivity. Measurements of fluorometric signal were performed with EnSight™ Multimode Plate Reader (PerkinElmer, Waltham, Massachusetts, United States), setting the excitation at 532 nm and the emission at 553 nm. MDA levels were drawn from the standard tuition and expressed as nmol/mL.

### Heme Oxygenase-1 Protein Level

HO-1 was detected in cell lysates by enzyme-linked immunosorbent assay (ELISA) kit (IMMUNOSET HO-1 human, ELISA development set, Enzo Life Sciences, Farmingdale, NY, United States), according to the manufacturer’s instructions. Briefly, samples were incubated with HO-1 capture antibody followed by washing steps and the addition of detection antibody for HO-1. The plate was incubated with streptavidin-conjugated HRP, and after a further wash, was incubated with TMB substrate and finally blocked with 1N HCl. After blanking the plate reader against the substrate, optical density was read at 450 nm using the EnSight™ Multimode Plate Reader (PerkinElmer, Waltham, Massachusetts, United States). Values of HO-1 concentration were plotted on a standard graph and expressed in ng/mL.

### Statistical Analysis

Data were evaluated on an iMac (Apple Computer, Cupertino, CA, United States) using GraphPad Prism 9.0 software (LA Jolla, CA, United States). Data are expressed as mean ± SD. The normal distribution of the variables was formally verified by Kolmogorov–Smirnov test. Data were analyzed using Student’s *t*-test for paired data. ANOVA was used to compare the quantitative variables. For all analyses, *p* < 0.05 was considered to indicate significant differences.

## Results

### p22^phox^ Protein Expression

**FIGURE 2:**
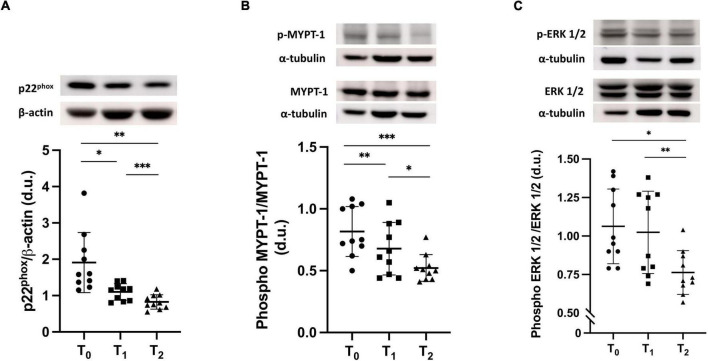
**(A)** p22^phox^ protein expression in FD patients before ERT (T_0_), after 12 months of ERT treatment (T_1_) and after 6 months of treatment with green tea on top of ERT (T_2_). The top part of the panel shows representative Western blot products of p22^phox^ protein expression of one patient. T_0_ vs. T_1_, *p = 0.01; T_0_ vs. T_2_, **p = 0.001; T_1_ vs. T_2_, ***p < 0.001. Repeated measures for one-way ANOVA, *p* = 0.003. **(B)** MYPT-1 phosphorylation state in FD patients at baseline (T_0_), at T_1_ and at T_2_. The top part of the panel shows representative Western blot products of p22^phox^ protein expression of one patient. T_0_ vs. T_1_ **p < 0.001; T_0_ vs. T_2_ ***p < 0.0001; T_1_ vs. T_2_, *p = 0.003. Repeated measures for one-way ANOVA, *p* < 0.001. **(C)** Phosphorylation state of ERK 1/2 in FD patients at T_0_, at T_1_ and at T_2_. The top part of the panel shows representative Western blot products of p22^phox^ protein expression of one patient. T_0_ vs. T_1_ p = ns_;_ T_0_ vs. T_2_ *p = 0.003; T_1_ vs. T_2_, **p = 0.002. Repeated measures for one-way ANOVA, *p* = 0.002.

Figure 2A shows that p22^phox^ protein expression was significantly decreased by ERT treatment (T_1_) compared to baseline (T_0_): 1.10 ± 0.23 d.u. vs. 1.91 ± 0.83 d.u. (*p* = 0.01), and further significantly decreased after GT treatment (T_2_) (0.83 ± 0.20 d.u.) compared to both baseline (*p* = 0.001) and T_1_ (*p* < 0.001).

### MYPT-1 Phosphorylation State

As shown in Figure 2B, the MYPT-1 phosphorylation state was higher at baseline (T_0_), significantly decreased after ERT (T_1_) [0.81 ± 0.20 d.u. vs. 0.68 ± 0.21 d.u. (*p* < 0.001)] and further significantly decreased with GT treatment (0.52 ± 0.11 d.u.) compared to both baseline and after 12 months of ERT (*p* < 0.0001 and *p* = 0.003, respectively).

### ERK 1/2 Phosphorylation State

ERK 1/2 phosphorylation was not statistically different between baseline (T_0_) and ERT treatment (T_1_) (1.06 ± 0.24 d.u. vs. 1.02 ± 0.27 d.u.), while it significantly decreased after 6 months of GT treatment (T_2_) (0.76 ± 0.14 d.u.) compared to both baseline (*p* = 0.003) and T_1_ (*p* = 0.002) (Figure 2C).

### Lipid Peroxidation

**FIGURE 3:**
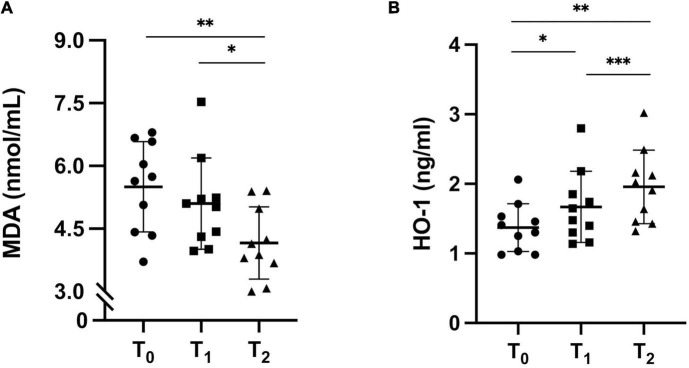
**(A)** Lipid peroxidation state. Fluorometric analysis of MDA concentration in FD patients before ERT (T_0_), after 12 months of ERT treatment (T_1_) and after 6 months of green tea on top of ERT (T_2_). T_0_ vs. T_1_ p = ns; T_1_ vs. T_2_, *p = 0.03; T_0_ vs. T_2_, **p = 0.005. ANOVA, *p* = 0.004. **(B)** HO-1 protein expression in FD patients at T_0_, at T_1_ and at T_2_. T_0_ vs. T_1_, *p = 0.04; T_0_ vs. T_2_, **p = 0.001; T_1_ vs. T_2_, ***p < 0.001. ANOVA, *p* = 0.001.

MDA levels, as a marker of lipid peroxidation, were unchanged after 12 months of ERT treatment (T_1_) compared to baseline (T_0_): 5.50 ± 1.08 vs. 5.10 ± 1.1 nmol/mL. Green tea supplementation (T_2_) induced a significant decrease of MDA plasma levels: 4.16 ± 0.86 nmol/mL, compared to baseline (*p* = 0.005) and T_1_ (*p* = 0.03) (Figure 3A).

### Heme Oxygenase-1 Protein Expression

HO-1 expression was increased by ERT treatment (T1) compared to baseline (T0): 1.67 ± 0.51 vs. 1.37 ± 0.34 ng/mL (*p* = 0.04). Adjuvant GT to ERT (T2) could improve the antioxidant defenses as HO-1 protein level further significantly increased: 1.96 ± 0.53 ng/mL, compared to both baseline (T0) and ERT treatment (T1) (*p* = 0.001 and *p* < 0.001, respectively) (Figure 3B).

## Discussion

This study shows that ERT treatment of FD patients and, in particular, the addition of the antioxidant treatment with GT, led to a reduction of OS in these patients, evaluated via a molecular biology approach. ERT treatment significantly reduced proteins related with OS and OS signaling, assessed by p22^phox^ protein expression, MYPT-1 phosphorylation state, and increased antioxidant defenses in terms of HO-1. Adjuvant GT treatment to ERT further significant decreased the reduction of the above-mentioned proteins related to OS and OS signaling and increased antioxidant defenses induced by ERT; GT treatment was also able to significantly reduce ERK 1/2 phosphorylation and MDA levels, on which ERT treatment had no impact.

FD is characterized by complex clinical features with symptoms overlapping other diseases, which may lead to delayed diagnosis and start of therapy. Although significant amelioration occurs with enzymatic therapy, especially in case of late diagnosis and treatment, ERT does not alter the natural course of the disease. This suggests that the adverse outcomes cannot be justified only by the accumulation of Gb3, and that additional therapies targeted at specific concomitant secondary mechanisms may impact the progression of cardiac, cerebrovascular, and gastrointestinal disease and the nephropathy occurring in FD patients.

OS, given its close relationship with endothelial dysfunction and cardiovascular-renal remodeling (12), is a main secondary process that takes place in FD patients (13).

In FD the presence of OS was first evaluated in terms of elevated markers of inflammation and oxidative damages (23, 24). In addition, our investigations on OS in FD patients showed not only increased levels of proteins related with OS and its signaling but also the association of increased OS with cardiac remodeling (13). This study evaluated the effect of an adjuvant antioxidant treatment with GT with ERT on biochemical pathways related to OS and OS signaling.

NADPH oxidases (NOXs), a group of membrane-associated enzymes, play a key role in the cellular fine tuning of redox balance. These enzymes catalyze the production of ROS through the association of different membrane and cytosolic subunits (25). Within the NOXs family, NOX2 is the isoform constitutively expressed in a variety of cell types and to be activated, requires the association of p22^phox^, which is an integral subunit of the final electron transport from NADPH to heme and molecular oxygen to generate the superoxide anion (26), and other three cytosolic subunits (25). In our previous report, we found increased ROS production associated with Gb3 accumulation in FD patients through the overexpression of p22^phox^ in FD patients compared to healthy subjects (13). The improvement of the deficient enzymatic activity of α-Gal A by ERT could significantly reduce the oxidative strain, as shown in this study. This reduction was further boosted by the scavenging activity of GT polyphenols given as an adjuvant to ERT.

The increased production of oxidants may influence the lipidic components of cellular membranes and affect the mediators of lipid signaling. Alongside lipid hydroperoxides, different aldehydes such as MDA, propanal, hexanal, and 4-HNE can be formed as secondary products (27). We have previously shown that treated FD patients are characterized by higher lipid peroxidation levels than healthy subjects (13), which was not reduced by the ERT treatment alone. The reduction of lipid peroxidation was instead documented in this study with the addition of adjuvant GT to ERT, further supporting a positive effect of the adjuvant antioxidant treatment in FD patients.

The activation of RhoA/Rho kinase (ROCK) pathway is linked with OS, atherosclerosis, and cardiovascular-renal remodeling (28, 29). The ROCK pathway promotes Ca^2+^ sensitization of smooth muscle contraction and the modulation of MLCP through inhibiting phosphorylation of MYPT-1 (30). In FD patients under ERT, the activation of ROCK was shown in terms of significantly higher phosphorylation status of its marker of activity, MYPT-1 (13). This study shows ROCK activation in untreated FD patients and demonstrates that ERT reduces ROCK activity, underlining the need for early diagnosis and prompt initiation of ERT and, most importantly, the adjuvant antioxidant treatment with GT further significantly reduced MYPT-1 phosphorylation, supporting the positive effect of an additive antioxidant treatment. Notably, EGCG, the most abundant catechin in GT, was shown to promote dephosphorylation of MYPT-1, indicating the reduction of ROCK activity, which is coupled with increased eNOS activity and NO production, to improve endothelial cells’ integrity (31, 32).

The intracellular signaling mediated by ERK 1/2 is also linked to OS. It is recognized as an effector for the hypertrophic response via phosphorylation of nuclear targets that, once activated, leads to transcriptional reprogramming and altered gene expression associated with hypertrophy, differentiation, and proliferation (33). We have documented the unexpected lower level of ERK1/2 phosphorylation in treated FD patients (13), which, however, has its rationale in the higher levels of cAMP found in FD patients induced by Gb3 and Lyso Gb3 (34). Indeed, in cardiomyocytes, the activation of ERK1/2 is under cAMP control that inhibits the ERK pathway via PKA activity (35). In this study, in fact, ERT treatment did not change ERK 1/2 phosphorylation state, while the additive treatment with GT led to a significant decrease of ERK 1/2 activation. Wu et al. explored the potential effect of EGCG in the suppression of ERK signaling and showed that EGCG decreased the protein levels of phospho-ERK 1/2 (36). In addition, EGCG was found to dose-dependently increase intracellular cAMP production (37). Therefore, although the reduction of Gb3 and Lyso Gb3 fostered by ERT treatment could result in cAMP reduction, with consequent increase of ERK phosphorylation, EGCG might compensate the ERT-mediated reduction of cAMP via stimulation of the cAMP/PKA pathway, finally leading to inhibition of the ERK 1/2, which is in agreement with the reduction of ERK 1/2 phosphorylation shown in this study in FD patients after GT treatment.

HO-1 is involved in the initial catabolism reaction of heme, producing CO, Fe^2+^, and biliverdin, which is further metabolized into bilirubin, a potent antioxidant. In the long term, HO-1 has also been shown to have anti-inflammatory and antiproliferative effects (38). Endogenous antioxidant defense in terms of HO-1, which we have previously shown to be reduced in treated FD patients compared to healthy subjects (13) was, in this study, increased by ERT treatment compared to baseline and further increased by adjuvant GT treatment, providing more evidence on the role of OS and OS reduction in addition to early initiation of ERT and antioxidant therapies in FD patients.

The results of this study provide further insights on the significance of OS in FD pathophysiology and the need to reduce OS in FD patients. A better understanding of specific molecular signaling in response to OS could provide additional interventions to contribute to slowing/halting the progress of cardiac and cerebrovascular disease and the nephropathy that occurs in FD patients.

In this regard, specific dietary interventions and bioactive compounds present in foods may interfere with specific OS pathways that induce tissue-damaging effects (39). To date, very few studies have investigated an antioxidant treatment in FD. One study showed that a 7-day treatment with vitamin E provided a moderate inhibitory effect on platelet aggregation in a cohort of FD patients (40). In another study, ascorbate, a potent antioxidant, was found to decrease cerebral hyperperfusion in FD patients on ERT (41). Recently, Kim et al. showed in an *in vitro* disease model of renal FD that treatment with the antioxidant GSH reduced OS and attenuated the structural alterations of the GLA-mutant kidney organoids compared to wild-type kidney organoids (42). Our data support and provide the mechanistic rationale to these studies which suggest that anti-inflammatory/antioxidative dietary patterns might be added to FD ERT treatment to contribute to slowing disease progression.

## Conclusion

Patients with FD are characterized by an oxidative imbalance, along with an impaired activation of antioxidant defenses, that should be taken into consideration in the management and treatment of FD. Although this study was limited by a small sample size because of the rare nature of FD and the overall short-term evaluation, our data highlight the antioxidant effect exerted by ERT itself, which is further amplified by the adjuvant antioxidant treatment with GT. This highlights the fundamental importance of early treatment for FD, while also underlining the likely positive effect of an adjuvant antioxidant treatment toward the reduction of OS. Studies with a larger sample size and a longer duration, in addition to confirming our results, could also provide insights to a possible link between specific GLA mutations and the severity of the oxidative profile, which might further highlight the importance of early ERT and antioxidant therapy to reduce OS, based on a patient-specific care plan.

## Data Availability Statement

The original contributions presented in this study are included in the article/supplementary material, further inquiries can be directed to the corresponding author/s.

## Ethics Statement

The studies involving human participants were reviewed and approved by the Ethical Committee of Padua University Hospital (CESC). The patients/participants provided their written informed consent to participate in this study.

## Author Contributions

GB and LAC: conceptualization and writing—original draft preparation. GB, VR, and FF: methodology. GB, VR, GC, and LAC: formal analysis. VR and LAC: writing—review and editing. LAC: supervision. VDV, PBA, and LS: bibliographic search. GC, NV, VDV, PBA, LS, and FF: patients management. All authors have read and agreed to the published version of the manuscript.

## Conflict of Interest

The authors declare that the research was conducted in the absence of any commercial or financial relationships that could be construed as a potential conflict of interest.

## Publisher’s Note

All claims expressed in this article are solely those of the authors and do not necessarily represent those of their affiliated organizations, or those of the publisher, the editors and the reviewers. Any product that may be evaluated in this article, or claim that may be made by its manufacturer, is not guaranteed or endorsed by the publisher.
